# Respiratory muscle training induces additional stress and training load in well-trained triathletes—randomized controlled trial

**DOI:** 10.3389/fphys.2023.1264265

**Published:** 2023-09-28

**Authors:** Tomasz Kowalski, Przemysław Seweryn Kasiak, Kinga Rebis, Andrzej Klusiewicz, Dominika Granda, Szczepan Wiecha

**Affiliations:** ^1^ Department of Physiology, Institute of Sport—National Research Institute, Warsaw, Poland; ^2^ 3rd Department of Internal Medicine and Cardiology, Medical University of Warsaw, Warsaw, Poland; ^3^ Department of Physical Education and Health in Biala Podlaska, Faculty in Biala Podlaska, Jozef Pilsudski University of Physical Education in Warsaw, Biala Podlaska, Poland; ^4^ Department of Nutrition Physiology and Dietetics, Institute of Sport—National Research Institute, Warsaw, Poland

**Keywords:** respiratory muscle training, respiratory muscles, training load, triathlon, triathlete

## Abstract

**Background:** Respiratory muscle training (RMT) has been investigated in the context of improved athletic performance and pulmonary function. However, psychophysiological costs of RMT remain understudied. Voluntary isocapnic hyperpnoea (VIH) and inspiratory pressure threshold loading (IPTL) are widely applied RMT methods. The main purposes of this study were to assess whether RMT induces additional load on well-trained triathletes and determine differences in RMT-induced load between sexes and applied methods.

**Materials and Methods:** 16 well-trained triathletes (*n* = 16, 56% males) underwent 6 weeks of VIH or IPTL program with progressive overload. Blood markers, subjective measures, cardiac indices, near-infrared spectroscopy indices, inspiratory muscle fatigue, and RMT-induced training load were monitored pre-, in and post-sessions. We used multiple ANOVA to investigate effects of sex, training method, and time on measured parameters.

**Results:** There were significant interactions for acid-base balance (*p* = 0.04 for sex, *p* < 0.001 for method), partial carbon dioxide pressure (*p* = 0.03 for sex, *p* < 0.001 for method), bicarbonate (*p* = 0.01 for method), lactate (*p* < 0.001 for method), RMT-induced training load (*p* = 0.001 for method for single session, *p* = 0.03 for method per week), average heart rate (*p* = 0.03 for sex), maximum heart rate (*p* = 0.02 for sex), intercostales muscle oxygenation (*p* = 0.007 for testing week), and intercostales muscle oxygenation recovery (*p* = 0.003 for testing week and *p* = 0.007 for method).

**Conclusion:** We found that RMT induced additional load in well-trained triathletes. Elicited changes in monitored variables depend on sex and training method. VIH significantly increased subjective training load measures. IPTL was associated with disbalance in blood gasometry, increase in lactate, and reports of headaches and dizziness. Both methods should be applied with consideration in high-performance settings.

## Introduction

Thorough evidence of the crucial role of respiratory strength, endurance, and fatigue in athletic performance emerged in the last decades (Boutellier et al., 1992; Volianitis et al., 2001; Romer et al., 2002). Systematic reviews of literature found that respiratory muscle training (RMT) may improve specific performance during time trials, constant load tests, and intermittent incremental tests (González-Montesinos et al., 2012; Illi et al., 2012; HajGhanbari et al., 2013), enhance respiratory muscle strength and endurance (González-Montesinos et al., 2012; HajGhanbari et al., 2013; Salesdo et al., 2016), and reduce respiratory fatigue, perceived breathlessness, and exertion during exercise in normoxia and hypoxia (Klusiewicz, n.d.; HajGhanbari et al., 2013; Álvarez-Herms et al., 2018). High levels of respiratory muscle fatigue are known to limit exercise performance among different sports, conditions, and populations (Mador and Acevedo, 1991; Amann et al., 2007; Verges et al., 2007). Moreover, cardiorespiratory fitness and sports performance depend on the athlete’s ventilation and lung function (Wiecha et al., 2023). RMT has received significant research attention beyond just the athletic community. It has been associated with improved balance and trunk control, endurance, pulmonary function, and quality of life in patients and the elderly (Keles et al., 2018; Ferraro et al., 2019; Zheng et al., 2021; Arslan et al., 2022). However, the traditional sport-specific training programs do not enhance the function of respiratory muscles (Coast et al., 1990; Eastwood et al., 2001; Klusiewicz et al., 2017), providing a rationale to introduce RMT.

The underpinning physiological mechanisms of the respiratory-related performance benefits are associated with delaying or attenuating of the respiratory metaboreflex (Dempsey et al., 2006; Witt et al., 2007; Illidi et al., 2023). The increased fatigue and accumulation of metabolites in respiratory muscles lead to a decrease in the blood flow of skeletal muscles and redirection of blood flow to respiratory muscles (Sheel et al., 2018). Consequently, the vasoconstriction in the exercising limbs may lead to increased local fatigue and limit performance (McConnell and Lomax, 2006; Romer et al., 2006). Therefore, due to improvement of mechanical efficiency and fatigue resistance of respiratory muscles, RMT is expected to constrain the accumulation of muscle metabolites triggered by exercise and mitigate its systemic repercussions (Sheel et al., 2018; Illidi et al., 2023). There are different RMT methods, techniques, devices, and protocols. The following study focuses on two methods that are widely used with athletes and general populations: voluntary isocapnic hyperpnoea (VIH), and inspiratory pressure threshold loading (IPTL). Both of them have been found effective (Illidi et al., 2023), however VIH is more associated with improving respiratory muscles endurance, and IPTL is more associated with improving respiratory muscles strength (McConnell and Romer, 2004; Illidi et al., 2023).

Limited research described VIH as time-consuming and physically demanding (McConnell, 2011; Bhammar et al., 2022) or reported sporadic complaints about side stitches and soreness of respiratory muscles (Boutellier and Piwko, 1992). Singular publications indicated that IPTL should not be considered as a significant training load (TL) and does not result in additional fatigue in trained individuals (McConnell and Lomax, 2006; Klusiewicz, 2007). RMT was previously investigated in regard to elicited performance changes, but not in regard to the psychophysiological cost. Monitoring and measuring TL enables coaches and support staff to adjust levels of physiological work to maximize performance, as well as reduce excessive levels of fatigue and the risk of injury or overtraining (Wing, 2018). However, to our knowledge, no study has examined the direct effects of RMT on TL measurements, and only a small number of studies evaluated the effects of RMT on stress- and fatigue-related indices (Foster et al., 2012; Sartorio et al., 2012; Briskey et al., 2020; Iqbal et al., 2023). The main purpose of this study was to comprehensively assess whether RMT puts extra load on athletes by assessing changes in blood markers, subjective measures, cardiac indices, and near-infrared spectroscopy (NIRS)-derived indices. The secondary aim was to determine if there are significant differences in RMT-induced load between sexes or the investigated training methods.

## Materials and methods

The study design was reviewed and approved by the Institute of Sport - National Research Institute Ethics Committee (approval no KEBN-23-78-TK). Informed written consent was obtained from all study participants. All procedures were carried out in accordance with the Declaration of Helsinki. The study was registered at ClinicalTrials.gov as NCT05936398. CONSORT guidelines for reporting parallel group randomized trials were applied (Schulz et al., 2011) (Supplementary Table S1).

### Participants

Sixteen well-trained triathletes (7 females and 9 males) volunteered to participate in the study in response to the invitation. The recruitment process occurred in January 2023 and February 2023. All the participants were classified in Tier 3 or Tier 4 according to the Participant Classification Framework (McKay et al., 2022), as highly trained or elite athletes. The inclusion criteria were: valid medical certificate to compete in triathlon, lack of previous experience with RMT, at least 6 years of triathlon training, average training volume over 12 h per week during last 6 weeks, performance caliber corresponding to at least medal placement during national multisport championship (any distance) during last 2 years. The exclusion criteria were: any chronic medical condition, any acute medical condition within last 3 months, any ongoing medication intake. Use of hormonal contraception and time since last menstruation were registered in females. All the study participants were in a similar training period (base training, after 10–14 weeks of structured training and few months before the competition season). The total required sample size was calculated with G* Power (version 3.1.9.2; Germany), with the level of significance set at *α* = 0.05, power (1 − β) = 0.80, and effect size ƒ = 0.42 (ANOVA with repeated measures, within-between interaction). According to the calculations, the required total sample size was 12 participants. To account for possible dropouts, 16 participants were recruited. All participants completed the study. Body composition was assessed with dual-energy x-ray absorptiometry using the Lunar Prodigy Pro DXA machine (GE Healthcare, Chicago, IL, United States). The participants’ characteristics at baseline are presented in Table 1. No statistically significant differences in any parameter were found between the groups (*p* > 0.05).

**TABLE 1:** Basic participant characteristics at baseline.

Variable/Group	VIH (n = 9)	IPTL (n = 7)
Age	30.2 ± 6.4	32.5 ± 4.3
Body mass	68.8 ± 10.42	67.3 ± 11.1
Body height	176.8 ± 10.9	173.3 ± 10.7
Body fat	14.6 ± 4.8	14.9 ± 4.5
Training experience	12.0 ± 3.6	15.6 ± 3.7
S-Index Test score	144.8 ± 41.7	151.4 ± 34.4

Values are mean ± standard deviation. VIH, voluntary isocapnic hyperpnoea; IPTL, inspiratory pressure threshold loading. Age is presented in [years], body mass is presented in [kg], body height is presented in [cm], body fat is presented as [percentage], training experience is presented in [years], S-Index Test score in [cmH2O]. In VIH, group were 4 (44.44%) females and 5 (55,56%) males, while in ITPL, group were 3 (42.86%) females and 4 (57.14%) males. All the participants were Caucasian. No statistically significant differences in any parameter were found between the groups (*p* > 0.05).

### Study design

The study was conducted as a randomized controlled trial with two parallel groups. Whereas participants and data collectors were aware of the allocated training method, the data analysts and laboratory technicians performing biochemistry assays were kept blinded to the allocation. The participants were assigned at random to either VIH or IPTL training group to perform RMT with progressive overload for 6 weeks. Initially, the participants performed S-Index Tests to measure inspiratory muscle strength (Silva et al., 2018). Subsequently, according to the results achieved, they were classified into corresponding pairs with the same sex. One individual in each pair was assigned to either VIH or IPTL group with a coin toss. Due to the odd number of each sex, unpaired participants were assigned to the training group with a coin toss.

The measurements were taken in week 1 (session 3 for VIH group and session 5 for the IPTL group), week 4 (session 14 for VIH and session 20 for the IPTL group), and week 6 (session 21 for VIH and session 30 for the IPTL group). All the measurements were taken in a laboratory setting. All the participants were adapted to laboratory settings due to their previous testing experiences. However, the investigators invited participants for the initial familiarization visit, when S-Index Tests were performed, RMT instructional presentations were delivered and a supervised training session has been completed. Then, three laboratory visits were required to perform the training session with multidimensional monitoring and investigators’ supervision. The participants were reminded weekly about executing prescribed training sessions via direct messages. During the study, 10 out of 16 participants were using TrainingPeaks App (TrainingPeaks LLC, Louisville, CO, United States) to plan and track training, including RMT sessions. Their personal accounts were attached to the investigators’ coaching account, allowing for constant training monitoring. All the participants were approached with regular follow-up at least once a week. According to self-declared reports, all of them meticulously followed the prescribed training program with a minimal number of minor changes to the planned workouts.

### Training protocols

VIH requires intentional, vigorous, paced ventilation for up to 30–40 min and uses partial rebreathing circuits to prevent hypocapnia. The training is based on hyperventilation at an intensity from 60% to 90% of maximal voluntary ventilation, with little or no external resistance (McConnell, 2011). The VIH group trained every second day with gradual progression based on session length and breathing frequency. Participants began with 3 min of exercise with a frequency of 20 breaths min^−1^ during the first session and added no more than 1 min or 2 breaths min^−1^ with each consecutive session. The course of the VIH training progression is presented in Table 2.

**TABLE 2:** The voluntary isocapnic hyperpnoea (VIH) group 6-week training protocol progression.

Session number	Session length	Breathing frequency	Session number	Session length	Breathing frequency
1	3	20	12	13	22
2	4	20	13	14	24
3	5	20	14	15	24
4	5	20	15	16	24
5	6	22	16	17	24
6	7	22	17	18	44
7	8	22	18	18	26
8	9	22	19	19	26
9	10	22	20	20	26
10	11	22	21	20	26
11	12	22			

Length of the session in [minutes], breathing frequency in [breaths·min^-1^].

IPTL uses breathing trainers with a spring-loaded inspiratory valve and an unloaded expiratory flap valve. During inspiration maneuvers participants must overcome the pressure load to open the valve and generate airflow, whereas no additional resistance during expiration is applied (Hart et al., 2001). Popular IPTL protocols are based on 30 full vital capacity inspirations from the residual volume level, against a resistance corresponding to 30%–50% of maximal inspiratory pressure (Illi et al., 2012; HajGhanbari et al., 2013). However, higher resistance may be successfully applied by elite endurance athletes (Klusiewicz et al., 2008). The IPTL group trained 5 days a week, twice a day, with at least 6 h break between sessions. The session consisted of 30 dynamic inspiratory maneuvers. The IPTL group was instructed to perform full vital capacity inspirations from residual volume level, against a resistance allowing them to perform 28–34 dynamic and powerful breaths. The IPTL group was instructed to increase the resistance periodically to account for training improvement.

### Testing design

**FIGURE 1:**
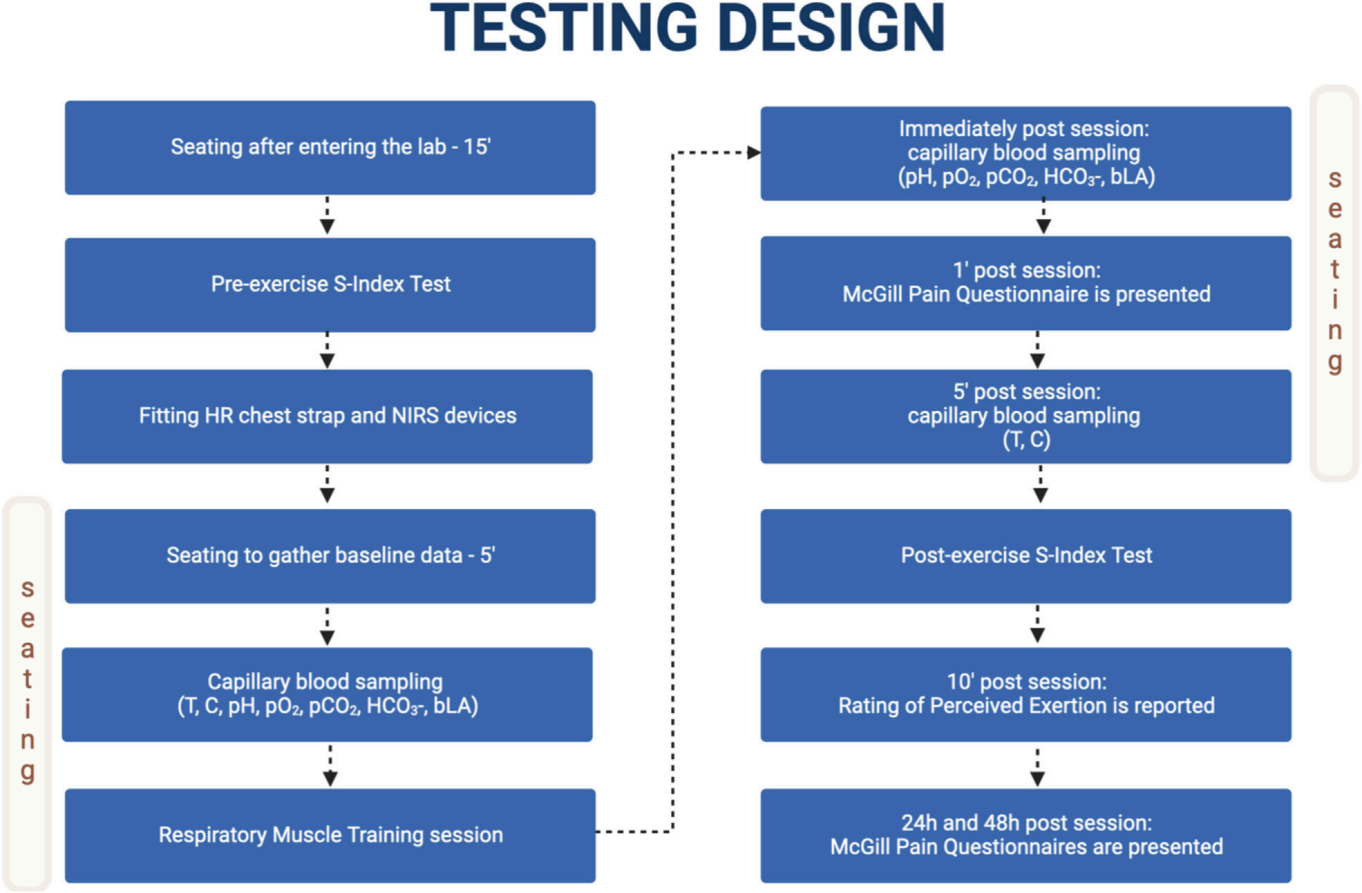
Testing design timeline. Adapted from “Flowchart Template”, by BioRender.com (2023). Retrieved from https://app.biorender.com/biorender-templates.

Testing was performed in the morning, between 8:30 and 10:30 a.m., to minimize potential physiological diurnal variation. The experimental data were gathered in the laboratory of the Department of Nutrition Physiology and Dietetics, Institute of Sport - National Research Institute, Warsaw, Poland. Repeatable testing conditions were provided with laboratory temperature varying from 20.7°C to 22.1°C, and humidity varying from 44% to 56%. The participants rested in a seating position for 15 min after entering the laboratory. At this point, McGill Pain Questionnaire (MPQ) was presented for the purpose of familiarization. Subsequently, the participant performed the S-Index Test. Then the HR chest strap and the near-infrared spectroscopy devices were fitted. Next, the participants sat for an additional 5 min to gather the baseline data for cardiac and near-infrared spectroscopy indices. All pre-session capillary blood samples were taken 1 min before the start of the RMT session. The session was performed in a seating position. VIH sessions consisted of 5 min of exercise in week 1, 15 min in week 4, and 20 min in week 6. The breathing frequency was 20 breaths min^−1^ in week 1, 24 breaths min^−1^ in week 3, and 26 breaths min^−1^ in week 6. Isocapnic BreathWayBetter device (Isocapnic Technologies Inc., Kelowna, Canada) with 6-L bags was used. IPTL sessions consisted of 2 min of dynamic and powerful breathing exercises with a Powerbreathe Plus - Medium Resistance device (POWERbreathe International Ltd., Southam, United Kingdom). The resistance was selected by the participants based on the instructions given before the start of training and corresponded to the resistance used during the regular IMT sessions in the current week. The average resistance used in week 1 was 39.50 cm H_2_O with an average of 31.43 breaths, in week 4 was 58.90 cm H_2_O with an average of 30.89 breaths, and in week 6 was 81.00 cm H_2_O with an average of 30.42 breaths. After the RMT sessions, the participant sat for another 5 min to gather the post-exercise data. Blood samples used for acid-base balance (pH), partial pressure of oxygen (pO_2_), partial pressure for carbon dioxide (pCO_2_), partial pressure for bicarbonate ion (HCO_3_^-^), and blood lactate (bLa) were collected immediately after cessation of the exercise and MPQ was presented 1 min after cessation of the exercise. Blood samples for cortisol (C) and testosterone (T) were collected 5 min after cessation of the exercise. The second S-Index Test was performed between minute 5 and minute 7 after cessation of the exercise. Rate of perceived exertion (RPE) was assessed 10 min after cessation of the exercise. The participants again answered MPQ after 24 and 48 h after the monitored RMT sessions. The detailed testing timeline is presented in Figure 1.

### Measured blood markers

Radiometer™ ABL90 FLEX blood gas analyzer (Radiometer Medical ApS, Brønshøj, Denmark) was used to measure pH, HCO_3-_, pO_2_, and pCO_2_. Super GL2 analyzer (Dr. Muller Geratebau GmbH, Freital, Germany) was used to measure bLa. Roche Cobas E 411 analyzer (Roche Diagnostics at F. Hoffmann-La Roche Ltd., Basel, Switzerland) was used to measure T and C with ElectroChemiLuminescence (ECL) technology for assay analysis. All the indices mentioned in this section were measured in capillary blood samples (45 µL for blood gas analysis, 20 µL for bLa analysis, and 300 µL for hormone analysis) taken from the fingertip before and after the monitored RMT sessions. Blood gas analysis and bLa analysis were performed immediately after RMT cessation. The samples for hormone measurements underwent centrifugation at 5,000 rpm for a duration of 10 min at a temperature of 4°C. Then, the serum was aliquoted and stored at a temperature of −20°C until the assays were performed during the next 24 h.

### Subjective measures

The Borg CR-10 Scale was presented to the participants and the assessment was made 10 min after the cessation of exercise (Scherr et al., 2013). Borg CR-10 Scale is widely used to assess RPE and prescribe training and monitor intensity (Thompson et al., 2013). In the past, all the participants utilized the scale to evaluate their athletic training. The session’s TL (sRPE) was calculated with a session RPE method (Foster et al., 2001). The method has demonstrated its validity, good reliability, and internal consistency across a variety of sports and physical activities, and has been utilized with individuals of different ages (children, adolescents, and adults) and skill levels (novices to experts), regardless of sex (Haddad et al., 2017). The average weekly TL induced by RMT (sRPEweek) was calculated with the assumption that VIH group performed RMT 3.5 times per week and IPTL group performed RMT 10 times per week. Calculations are presented below:

sRPE=RPE x length of RMT session in minutes

sRPEweek=sRPE x average number of RMT sessions during the week

MPQ is based on self-reported measures of pain and it assesses both the quality and intensity of subjective pain (Melzack, 1975). MPQ was selected due to its validated usefulness in pain research, including delayed onset muscle soreness (Melzack, 1975; Cleather and Guthrie, 2007), and psychometric properties (Graham et al., 1980). The Polish adaptation of the questionnaire developed by Kazimierz Sedlak was presented to the participants. The instrument comprises 78 adjectives categorized into twenty subcategories, with ten representing the sensory dimension of pain, five representing the affective dimension, one representing the evaluative dimension, and four miscellaneous subcategories. Participants were asked to choose words that best described their pain, resulting in a collection of descriptors that characterize an individual’s pain when the MPQ is completed. Each word in the MPQ is assigned a numerical value on a 0 to 5 scale based on its relative strength compared to predetermined anchor words. The total scale values of all the chosen words and the descriptive summary of words chosen most often were included in the analysis. Additionally, participants reported accompanying symptoms with an optional MPQ section, and the descriptive summary of symptoms chosen most often was included in the analysis.

### Cardiac indices

Heat rate (HR) and Heart Rate Recovery (%HRR) were monitored during and after RMT sessions with a Polar H10 chest strap (Polar Electro Oy, Kempele, Finland) paired with HRV Logger app (A.S.M.A. B.V., Marco Altini, Version 5.1.0, dowloaded from Mac App Store on 3 Feb 2022). %HRR was calculated as below.

$$\%\text{HRR }\left(\%\right)=\left(\text{HR at}1\;\min\text{afterthe cessation of exercise}/\text{maximal HR during RMT session}\right)\times 100$$

### NIRS-derived indices

Two NIRS monitors (Moxy monitors; Fortiori Design LLC, Hutchinson, MN, United States) were used to measure local muscle oxygenation (SmO2). The first device was fitted on the right vastus lateralis, approximately 13 ± 2 cm above the proximal border of the patella and 4 ± 2 cm lateral to the midline of the thigh. The skinfold tissue thickness was measured in the same place with Harpenden Skinfold Caliper (Baty International, Burgess Hill, West Sussex, United Kingdom). The obtained values (7.39 ± 2.97 mm) allowed for physiologically credible SmO_2_ measurements (McManus et al., 2018). The second monitor was fitted on the right intercostales, at the seventh intercostal space, at the anterior axillary line. Dark dynamic tape of 7.5 cm width was used to provide a repeatable testing environment and hypoallergenic skin tape was used to fix devices to the skin. A computation window of 2 s was used. SmO_2_ changes during RMT session (ΔSMO_2_B) and SmO_2_ recovery after RMT session (ΔSMO_2_A) were recorded to assess exercise intensity (Contreras-Briceño et al., 2022) as below:

$$\Delta {\text{SMO}}_{2}B={\text{average SMO}}_{2}\text{ concentration during }2‐\text{minute period before RMT}‐{\text{minimal SMO}}_{2}\text{ concentration during RMT session}$$

$$\Delta {\text{SMO}}_{2}A={\text{maximal SMO}}_{2}\text{ concentration during }2‐\text{minute period after RMT}‐{\text{minimal SMO}}_{2}\text{ concentration during RMT session}$$

### Other measured variables

Inspiratory muscle fatigue (IMF) was measured by performing pre- and post-training sessions S-Index Tests. S-Index Test is a dynamic spirometry assessment used to evaluate inspiratory muscle strength (Silva et al., 2018). POWERbreathe K5 device was used (POWERbreathe International Ltd., Southam, United Kingdom).

TL produced by RMT (%TTL) was assessed in the context of overall TL and calculated as below:

$$\%\text{TTL}=\left(\text{sRPEweek produced by RMT}/\text{overall weekly sRPE produced by all training sessions}\right)\times 100$$

### Statistical analysis

The normality of the distribution was tested visually with Q-Q plot figures and the Shapiro-Wilk test.

Independent t-tests were used to assess the differences in participant characteristics between groups. The main effects for type of training method, time of measurement (testing week), sex, and interaction of main effects were assessed by repeated-measures analysis of variance (ANOVA) with additional covariate assessment for training experience, age, and somatic indices, and body composition. Additionally, homogeneity was assessed with Levene’s test, and sphericity of variance using Mauchly’s test for spherical Greenhouse-Geisser correction, was applied. In significant main effects, a *post hoc* assessment was performed using Bonferroni correction. Significance was set at *p* < 0.05. Results are presented as mean and standard deviation. The effect size was determined by partial eta squared (ηp^2^) and omega squared (ω^2^) (Sullivan and Feinn, 2012; Lakens, 2013).

All statistical analyses were performed using the JASP Team statistical package JASP (Amsterdam, Netherlands, Version 0.17.2).

## Results

### Blood markers

Measurements for blood markers stratified by testing week, sex, and training method are presented in Table 3.

**TABLE 3:** Differences in blood markers for respiratory muscle training.

Testing week	Sex	Training method	ΔpH†*	ΔpCO_2_†*	ΔpO_2_	ΔHCO_3-_†	ΔbLa†	ΔC	ΔT
1st	F	VIH	0.14 (0.19)	−3.85 (3.62)	23.09 (13.00)	−1.73 (0.98)	−18.96 (9.08)	1.29 (5.29)	−0.46 (6.38)
		IPTL	1.53 (0.16)	−27.39 (2.95)	5.73 (19.14)	−6.82 (1.32)	38.27 (36.23)	−6.44 (3.39)	−5.53 (15.14)
	M	VIH	−0.16 (0.29)	4.10 (6.49)	7.28 (8.17)	0.44 (1.65)	−2.55 (17.18)	−3.47 (6.83)	8.33 (13.21)
		IPTL	1.14 (0.50)	−21.44 (9.58)	15.19 (19.42)	−4.77 (4.23)	23.90 (18.52)	−3.93 (4.10)	7.81 (4.77)
4th	F	VIH	−0.03 (0.43)	−10.74 (7.86)	23.87 (7.15)	1.26 (2.73)	−37.55 (11.26)	−8.84 (8.63)	2.46 (3.47)
		IPTL	1.57 (0.55)	−30.66 (10.04)	15.43 (10.51)	−10.00 (4.00)	34.16 (38.31)	−4.55 (1.81)	19.94 (22.36)
	M	VIH	−0.03 (0.51)	2.32 (10.34)	9.12 (16.48)	1.00 (2.80)	−21.81 (21.67)	−3.36 (13.40)	12.82 (12.52)
		IPTL	0.71 (0.42)	−12.74 (9.81)	8.15 (5.76)	−2.61 (4.31)	8.68 (20.60)	2.79 (15.94)	9.19 (9.41)
6th	F	VIH	−0.03 (0.23)	−0.25 (2.54)	16.41 (11.27)	−0.80 (0.92)	−7.28 (15.46)	−1.41 (8.80)	2.52 (28.70)
		IPTL	1.57 (0.87)	−23.53 (22.83)	19.77 (27.15)	−2.22 (17.80)	26.84 (3.67)	−8.52 (6.04)	4.32 (38.55)
	M	VIH	0.03 (0.39)	0.57 (8.33)	11.88 (13.64)	0.76 (3.19)	−7.47 (33.93)	−7.24 (8.95)	2.86 (8.61)
		IPTL	0.74 (0.50)	−15.90 (10.12)	17.95 (18.09)	−4.87 (3.91)	32.52 (44.69)	−2.54 (7.10)	1.76 (15.84)

Abbreviations: ΔpH, difference in acid-base balance before and after session; ΔpCO_2_, difference in partial pressure for carbon dioxide before and after session [percentage]; ΔpO_2_, difference in partial pressure of oxygen before and after session [percentage]; ΔpHCO_3_^-^, difference bicarbonate ion concentration before and after session [percentage]; ΔbLA, difference in blood lactate concentration before and after session [percentage]; ΔC, difference in blood cortisol concentration before and after session [percentage]; ΔT, difference in blood testosterone concentration before and after session [percentage]; SD, standard deviation; F, females; VIH, voluntary isocapnic hyperpnoea; IPTL, inspiratory pressure threshold loading; M, males. Athletes underwent respiratory muscle training three times: in first, fourth and sixth week. Data are presented as mean with (standard deviation). Significant interactions between variables and training method were marked with †, while significant interactions with sex were marked with *.

There was a statistically significant interaction between ΔpH and sex (F (1, 2) = 5.428, *p* = 0.04, η_p_^2^ = 0.31) or training method (F (1, 2) = 55.438, *p* < 0.001, η_p_^2^ = 0.82). The mean value of ΔpH was significantly higher for IPTL than VIH (Mean Difference = −1.23, SE = 0.17, d = −2.78) and for females than males (Mean Difference = 0.38, SE = 0.17, d = 0.87).

There was also a statistically significant interaction between ΔpCO_2_ and sex (F (1, 2) = 6.45, *p* = 0.03, η_p_^2^ = 0.35, ω^2^ = 0.15) or training method (F (1, 2) = 34.77, *p* < 0.001, η_p_^2^ = 0.74, ω^2^ = 0.68). The mean value of ΔpCO_2_ was significantly higher for VIH than IPTL (Mean Difference = 20.64, SE = 3.50, d = 2.17) and for males than females (Mean Difference = −8.89, SE = 3.50, d = −0.94).

Another significant interaction has been found between ΔpHCO3- and the training method (F (1, 2) = 8.17, *p* = 0.01, η_p_^2^ = 0.41, ω^2^ = 0.22). The mean value of ΔpHCO_3_^-^ was significantly higher for VIH than IPTL (Mean Difference = 4.95, SE = 1.73, d = 0.97).

The last significant interaction occurs between ΔbLa and the training method (F (1, 2) = 39.37, *p* < 0.001, η_p_^2^ = 0.77, ω^2^ = 0.60). The mean value of ΔbLa was significantly higher for IPTL than VIH (Mean Difference = −43.33, SE = 6.91, d = −1.72).

### Subjective measures

Subjective measures stratified by testing week, sex, and training method are presented in Table 4.

**TABLE 4:** Differences in subjective measures for respiratory muscle training.

Testing week	Sex	Training method	sRPE†	sRPEweek†	MPQI	MPQ24	MPQ48
1st	F	VIH	16.25 (8.54)	56.88 (29.89)	2.00 (2.16)	0.00 (0.00)	0.00 (0.00)
		IPTL	6.67 (1.16)	66.67 (11.55)	8.00 (7.94)	0.00 (0.00)	0.00 (0.00)
	M	VIH	15.00 (7.07)	52.50 (24.75)	2.40 (2.30)	0.00 (0.00)	0.00 (0.00)
		IPTL	8.50 (4.12)	85.00 (41.23)	4.25 (7.18)	0.00 (0.00)	0.00 (0.00)
4th	F	VIH	71.25 (43.08)	249.38 (150.80)	0.00 (0.00)	0.00 (0.00)	0.00 (0.00)
		IPTL	8.00 (3.46)	80.00 (34.64)	0.00 (0.00)	0.00 (0.00)	0.00 (0.00)
	M	VIH	57.00 (22.25)	199.50 (77.87)	0.00 (0.00)	0.00 (0.00)	0.00 (0.00)
		IPTL	7.50 (2.52)	75.00 (25.17)	0.00 (0.00)	0.00 (0.00)	0.00 (0.00)
6th	F	VIH	70.00 (52.92)	245.00 (185.20)	0.00 (0.00)	0.00 (0.00)	0.00 (0.00)
		IPTL	6.67 (4.62)	66.67 (46.19)	0.00 (0.00)	0.00 (0.00)	0.00 (0.00)
	M	VIH	72.00 (41.47)	252.00 (145.16)	0.20 (0.45)	0.00 (0.00)	0.00 (0.00)
		IPTL	11.50 (4.44)	115.00 (44.35)	0.00 (0.00)	0.00 (0.00)	0.00 (0.00)

Abbreviations: sRPE, declared rating of perceived exertion for one session; sRPEweek, sum of rating of perceived exertion for all respiratory muscles training sessions in a week; MPQI, total scale values of all the chosen words in McGill Pain Questionnaire declared immediately after session; MPQ24, total scale values of all the chosen words in McGill Pain Questionnaire declared 24 h after session; MPQ48, total scale values of all the chosen words in McGill Pain Questionnaire declared 48 h after session; SD, standard deviation; F, females; VIH, voluntary isocapnic hyperpnoea; IPTL, inspiratory pressure threshold loading; M, males. Athletes underwent respiratory muscle training three times: in first, fourth and sixth week. Data are presented as mean with (standard deviation). Significant interactions between variables and training method were marked with †.

There was a statistically significant interaction between sRPE and training method (F (1, 2) = 16.81, *p* = 0.001, η_p_^2^ = 0.58, ω^2^ = 0.38). The mean value of sRPE was significantly higher for VIH than IPTL (Mean Difference = −42.11, SE = 10.27, d = 1.65).

There was also a statistically significant interaction between sRPEweek and training method (F (1, 2) = 6.48, *p* = 0.03, η_p_^2^ = 0.35, ω^2^ = 0.17). The mean value of sRPEweek was significantly higher for IPTL than VIH for first round and lower for IPTL than VIH for second and third round (Mean Difference = 94.49, SE = 37.11, d = 1.03).

The following pain descriptors in MPQI were chosen more than once. For IPTL during the first round: boring (1 time for male and 1 time for female), pressing (1 time for male and 1 time for female), tight (1 time for male and 1 time for female). For VIH during the first round: tender (2 times for male and 1 time for female), pressing (1 time for male and 1 time for female). For VIH during the third round: tiring (2 times). No pain descriptors were chosen in MPQ24 and MPQ48. The following accompanying symptoms in MPQI were chosen more than once. For IPTL during the first round: headache (1 time for males and 3 times for females), dizziness (1 time for males and 3 times for females). For IPTL during the second round: headache (3 times for females), dizziness (2 times for females). For IPTL during the third round: headache (3 times for females), dizziness (2 times for females). No accompanying symptoms were chosen in MPQ24 and MPQ48.

### Cardiac indices

Cardiac indices stratified by testing week, sex, and training method are presented in Table 5.

**TABLE 5:** Differences in cardiac indices for respiratory muscle training.

Testing week	Sex	Training method	HRavg*	HRmax*	%HRR
1st	F	VIH	79.00 (7.35)	86.25 (7.32)	74.26 (8.91)
		IPTL	107.33 (22.90)	119.68 (22.81)	54.08 (8.12)
	M	VIH	76.00 (4.72)	84.20 (6.91)	71.84 (8.46)
		IPTL	77.75 (17.23)	83.75 (20.53)	70.91 (11.46)
4th	F	VIH	87.00 (16.83)	93.25 (20.04)	82.52 (6.08)
		IPTL	92.00 (4.58)	108.68 (7.02)	64.96 (6.58)
	M	VIH	84.20 (15.96)	92.00 (15.81)	78.49 (5.55)
		IPTL	69.50 (16.98)	75.25 (19.76)	76.35 (17.02)
6th	F	VIH	82.00 (6.98)	94.25 (8.26)	72.04 (5.41)
		IPTL	95.00 (7.00)	102.00 (5.00)	69.12 (6.71)
	M	VIH	80.40 (10.36)	87.80 (11.26)	84.27 (7.95)
		IPTL	76.25 (16.66)	82.00 (18.06)	76.94 (16.66)

Abbreviations: HRavg, average heart rate during session [beats·min^-1^]; HRmax, maximal heart rate during session [beats·min^-1^]; %HRR, post-exercise heart rate recovery [percentage]; SD, standard deviation; F, females; VIH, voluntary isocapnic hyperpnoea; IPTL, inspiratory pressure threshold loading; M, males. %HRR, was calculated as (heart rate at first minute after the cessation of exercise/maximal heart rate during session) × 100. Athletes underwent respiratory muscle training three times: in first, fourth and sixth week. Data are presented as mean with (standard deviation). Significant interactions between variables and sex were marked with *.

There was a statistically significant interaction between HRavg and sex (F (1, 1.18) = 5.71, *p* = 0.03, η_p_^2^ = 0.32, ω^2^ = 0.15). The mean value of HRavg was significantly higher for females than males (Mean Difference = −13.04, SE = 5.46, d = 0.97).

Another significant interaction has been found between HRmax and sex (F (1, 1.39) = 6.81, *p* = 0.02, η_p_^2^ = 0.36), ω^2^ = 0.18). The mean value of HRmax was significantly higher for females than males (Mean Difference = 16.51, SE = 6.33, d = 1.12).

### NIRS-derived indices

**FIGURE 2:**
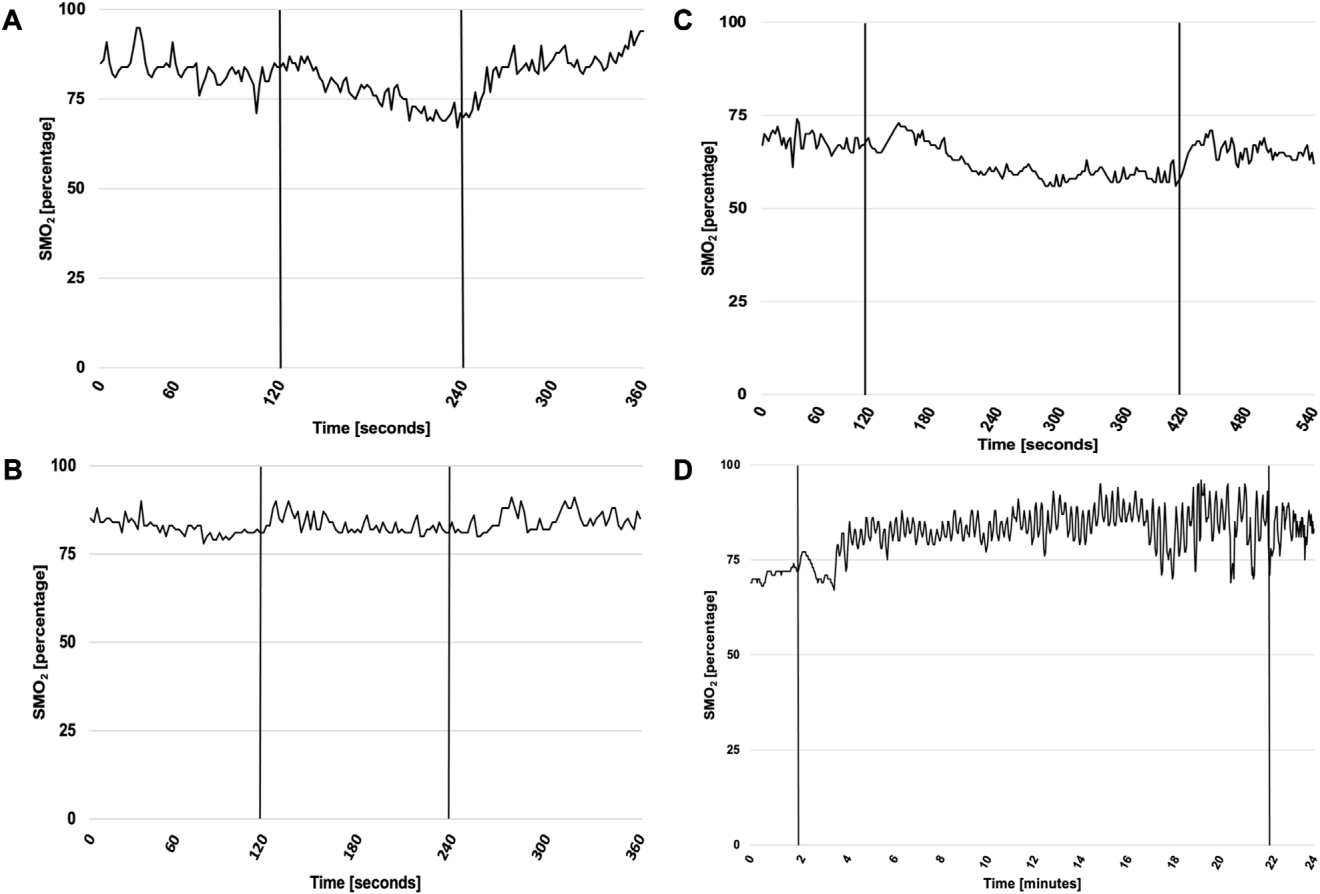
Exemplary time course of muscle oxygenation for intercostales during RMT in first and sixth testing week. Abbreviations: SMO_2_, local muscle oxygenation. **(A)** presents SMO_2_ for ITPL in first week, **(B)** for ITPL in sixth week, **(C)** for VIH in first week and **(D)** for VIH in sixth week. ITPL lasted 2 min both in first and sixth week, while VIH lasted 5 min in first week and 20 min in sixth week. Vertical lines represent start and cessation of RMT. The exemplary plots (male participant for ITPL, female participant for VIH) illustrate time course of SMO_2_ changes for intercostales. Throughout the monitored sessions, SMO_2_ usually increased during first respiratory maneuvers during all monitored sessions. In first testing week muscle deoxygenation was usually observed, however in sixth testing week no muscle deoxygenation was noticed during RMT for intercostales, suggesting RMT-related adaptation of muscle function.

NIRS-derived indices stratified by testing week, sex, and training method are presented in Table 6. Exemplary time course of muscle oxygenation for intercostales is presented in Figure 2.

**TABLE 6:** Differences in NIRS-derived indices for respiratory muscle training.

Testing week	Sex	Training method	ΔSMO_2_BI‡	ΔSMO_2_BV	ΔSMO_2_AI†‡	ΔSMO_2_AV
1st	F	VIH	6.65 (27.97)	10.47 (28.31)	12.75 (12.18)	7.75 (9.64)
		IPTL	31.71 (16.48)	8.82 (24.40)	−22.33 (4.62)	−5.67 (17.24)
	M	VIH	−24.78 (24.28)	−10.65 (15.90)	21.03 (15.13)	16.40 (5.90)
		IPTL	5.67 (45.77)	4.39 (17.82)	2.00 (26.73)	−5.50 (20.66)
4th	F	VIH	−7.73 (6.06)	−8.37 (7.09)	14.25 (3.30)	14.50 (9.04)
		IPTL	−2.49 (0.90)	−2.82 (1.16)	24.93 (8.97)	12.64 (5.51)
	M	VIH	−15.39 (14.42)	−2.32 (11.33)	16.80 (4.60)	13.40 (5.41)
		IPTL	−8.33 (8.35)	−7.49 (5.40)	12.25 (6.40)	11.00 (1.63)
6th	F	VIH	−24.60 (21.73)	−4.87 (7.34)	28.50 (13.30)	16.00 (7.07)
		IPTL	−14.46 (14.40)	−3.95 (2.58)	24.67 (10.12)	14.33 (5.51)
	M	VIH	−19.48 (11.25)	−3.23 (2.50)	25.00 (12.17)	12.20 (3.49)
		IPTL	−15.54 (10.85)	−4.21 (7.15)	18.50 (8.43)	14.00 (7.70)

Abbreviations: ΔSMO2BI, difference in local muscle oxygen saturation before session for intercostales [percentage]; ΔSMO2BV, difference in local muscle oxygen saturation before session for vastus lateralis muscle [percentage]; ΔSMO2AI, difference in local muscle oxygen saturation after session for intercostal muscles [percentage]; ΔSMO2AV, difference in local muscle oxygen saturation after session for vastus lateralis muscle [percentage]; SD, standard deviation; F, females; VIH, voluntary isocapnic hyperpnoea; IPTL, inspiratory pressure threshold loading; M, males. Athletes underwent respiratory muscle training three times: in first, fourth and sixth week. Data are presented as mean with (standard deviation). Significant interactions between variables and training method were marked with †, while significant interaction with testing week were marked with ‡.

There was a statistically significant interaction between ΔSMO_2_B for intercostales and testing week (F = 6.27, *p* = 0.01, η_p_^2^ = 0.34, ω^2^ = 0.21). The mean value of ΔSMO_2_B was decreasing with each testing session (Mean Difference = −7.09 SE = 3.95, d = 0.63 between first and fourth week, Mean Difference = −6.91 SE = 3.95, d = 0.28 between fourth and sixth week).

There was also a statistically significant interaction between ΔSMO_2_A for intercostales and testing week (F = 9.57, *p* = 0.003, η_p_^2^ = 0.44, ω^2^ = 0.35). The mean value of ΔSMO_2_A was increasing with each testing session (Mean Difference = 14.97 SE = 4.90, d = 1.20 between first and fourth week, Mean Difference = 5.86 SE = 4.90, d = 0.47 between fourth and sixth week).

Another significant interaction has been found between ΔSMO_2_A for intercostales and method (F (1, 2) = 10.79, *p* = 0.007, η_p_^2^ = 0.47, ω^2^ = 0.27). The mean value of ΔSMO_2_A was higher for VIH than IPTL for first and third round and lower for VIH than IPTL for second round (Mean Difference = 8.89 SE = 2.71, d = 0.72).

### Other measured variables

Other measured variables stratified by testing week round, sex, and training method are presented in Table 7.

**TABLE 7:** Differences in other measured variables for respiratory muscle training.

Testing week	Sex	Training method	%TTL	%ΔS-index
1st	M	VIH	0.83 (0.41)	1.64 (12.30)
		IPTL	1.69 (0.28)	4.15 (7.22)
	F	VIH	1.37 (1.01)	−2.17 (4.50)
		IPTL	1.69 (0.71)	2.21 (4.63)
4th	M	VIH	3.52 (2.08)	1.61 (2.76)
		IPTL	1.99 (0.67)	3.47 (3.62)
	F	VIH	5.14 (3.47)	−0.66 (3.59)
		IPTL	1.57 (0.76)	−0.35 (1.15)
6th	M	VIH	3.50 (2.67)	1.57 (5.84)
		IPTL	1.63 (0.95)	3.03 (4.06)
	F	VIH	6.61 (5.51)	4.63 (8.77)
		IPTL	2.29 (0.70)	2.97 (4.19)

Abbreviations: %TTL, part of total training load induced by respiratory muscle training [percentage]; ΔS-Index, difference in S-Index Test before and after session [percentage]; SD, standard deviation; F, females; VIH, voluntary isocapnic hyperpnoea; IPTL, inspiratory pressure threshold loading; M, males. %TTL, was calculated as (sRPE, for RMT/sRPE, for all activities) × 100. Athletes underwent respiratory muscle training three times: in first, fourth and sixth week. Data are presented as mean with (standard deviation).

## Discussion

To our knowledge, this is the first study to assess comprehensively psychophysiological cost of RMT. The main objective of our study was to assess whether RMT puts extra load on athletes and to determine if there are significant differences in RMT-induced load between sexes or applied training methods. Using multiple objective and subjective exertion measures we were able to capture multidimensional fatigue, stress, and TL induced by popular RMT training methods. As in many studies, the magnitude of observed changes significantly varies between the participants. However, inter-individual variability exhibits similar level in both investigated methods. We found that VIH and IPTL contribute to overall TL, therefore should be applied with consideration. The investigated methods differ in eliciting changes in monitored variables. Moreover, RMT induced larger changes in blood gasometry and cardiac indices in females rather than in males.

### Blood markers

Blood gas analysis showed larger post-RMT differences in females rather than in males, and VIH induced smaller changes in blood gasometry compared to IPTL. In literature, RMT was associated with mild hypocapnia (McConnell and Griffiths, 2010) and respiratory alkalosis due to hyperventilation resulting in pH and pO_2_ increase and pCO_2_ decrease (Djarova et al., 1986; Brown et al., 2010). However, according to our results, the extent of blood gasometry changes elicited by RMT may depend on the method of RMT or sex. Since IPTL training protocols are usually based on multiple (10–12) sessions per week, significant changes in blood gasometry also occur multiple times per week. However, the short- or long-term influence of such changes in athletes is an understudied area and the relevance of the aforementioned findings require further investigation. The differences in RMT-induced blood gasometry changes between males and females may be associated with functional consequences of sex-differences in the structure of respiratory system. The difference in lungs and rib-cage shape between the sexes, and proportionally smaller airways and lungs in women than man have been reported in the literature. In consequence, lungs’ volume and pressure, flow characteristic and regulation of blood gas homeostasis exhibit different patterns in man and women during exercise (Molgat-Seon et al., 2018; Archiza et al., 2021). ΔbLa was significantly higher for IPTL than VIH, with increased bLa after IPTL and decreased bLa after VIH. It confirms the theory that respiratory muscles may act as net consumers of lactate during recovery from intense exercise and the possibility of a decrease in bLa concentration after RMT (Spengler et al., 1999; Chiappa et al., 2008). Although it may seem otherwise, our findings that IPTL increased bLa concentration are not contrary to findings from Chiappa et al., 2008, where the bLa decreased after IPTL. In the mentioned study IPTL was introduced after intense exercise, whereas in our investigations we operated on relatively lower bLa concentration. Despite the potential usefulness of both methods, we speculate that VIH may be more appropriate as an active recovery protocol compared to IPTL. Implementing RMT as a recovery protocol requires further investigation with emphasis on the choice of method, timing, length, and intensity of RMT. No significant differences for ΔC and ΔT between groups were found. However, we noticed a trend for acute pre- and post-session changes in C and T concentration in both groups. Our results are inconclusive about the direction and extent of induced changes in first and second rounds. However, in third round, after a 6-week training program and improved RMT status, all groups noted decrease in C and increase in T. Therefore, we do not consider observed hormonal changes as noteworthy fatigue or stress effects after RMT.

### Subjective measures

sRPE was significantly higher for VIH than IPTL and the difference increased with the applied progressive overload. In IPTL, progressive overload comes from increase in resistance, but there is no increase in duration of exercise. In VIH, progressive overload comes from both increases in breathing frequency and duration of exercise. Consequently, as VIH training sessions significantly increased in duration from 5 min in first week to 20 min in sixth week, the sRPE also significantly increased. sRPE calculated for IPTL was more stable since there was no increase in duration and perceived exertion associated with increased resistance. We conclude that the main sRPE contributor during RMT is duration, rather than intensity coming from resistance or breathing frequency. Hence, the RMT based on longer exercise duration, such as VIH, tend to generate higher sRPE scores. However, since the number of sessions per week was different, sRPEweek was higher for IPTL than VIH in first week and lower for IPTL than VIH in fourth and sixth week. MPQI scores showed few measures of pain with very limited exertion and high individual variation. MPQ24 and MPQ48 results showed that no measures of pain were reported 24 and 48 h after the RMT session. Based on the reported measures, RMT did not deliver significant acute pain or heavy fatigue resulting in delayed onset muscle soreness. Noteworthy, headache or dizziness were reported in MPQI by many participants from the IPTL group in all three monitored training rounds. Both symptoms may be explained by blood gasometry changes associated with IPTL, as described before, which may be interpreted as hypercapnia. Hypercapnia causes vasodilation of cerebral arteries and contributes to an increase in cerebral blood flow (Ainslie and Duffin, 2009). As a result, elevated intracranial pressure may lead to headaches and dizziness (Ramadan, 1996). Both gasometry changes and the mentioned symptoms were larger for females than males. The latter may be associated with higher cerebral blood flow in females (Rodriguez et al., 1988), which potentially overlaps with RMT effects. The symptoms tend to lessen in frequency and severity with the progression of the training program, which confirms the findings of McConnell (McConnell, 2011). However, they were still reported by study participants in the third round, after almost 6 weeks of regular RMT. The reported dizziness decreased to a larger extent than the reported headache. Interestingly, the reduction of pain measures and accompanying symptoms with time was not associated with the reduction in blood gasometry changes. Overall, the subjective negative effects of RMT sessions are temporary rather than chronic.

### Cardiac indices

Both HRavg and HRmax were significantly higher for females than males. Interestingly, higher HR values were not reflected in sRPE assessment. Larger cardiac response to RMT may be associated with sex-differences in the structure and function of respiratory system, as pulmonary function and exercise capacity differ in females and males. Therefore, the increased work of breathing during exercise may lead to higher cardiac load in females (Harms, 2006; Archiza et al., 2021). It may suggest that women are predisposed to increased respiratory muscle fatigue, however after exercising to exhaustion the diaphragm fatigue is smaller in woman then man (Guenette et al., 2010), possibly due to extra-diaphragmatic inspiratory muscle recruitment (Molgat-Seon et al., 2018). This may partially explain higher HR values, since more muscles are engaged in the work of breathing. We also speculated that the level of motivation and engagement might have been lower in male compared to female participants, resulting in lower HR. Such interpretation may indicate possible challenges of implementing RMT in real-life environment.

### NIRS-derived indices

No significant differences in ΔSMO_2_BV, and ΔSMO_2_AV were noted in the context of training methods, sex, and testing week. There were significant differences in ΔSMO_2_BI between testing week and ΔSMO_2_AI between testing week and methods. Noteworthy patterns in local muscle oxygen saturation were observed. The extent of muscle deoxygenation during RMT became smaller with time, resulting in negative ΔSMO_2_BI and ΔSMO_2_BV for fourth and sixth week. That means that after the initial daptation period, local oxygenation tends to increase during RMT session. The increase in oxygenation was higher in intercostales compared to vastus lateralis. The difference between muscle groups is consistent with the observation of Espinoza-Ramirez et al. (2023), who showed decreased deoxygenation of intercostales during exercise after RMT program, with no effect of vastus lateralis oxygenation (Espinosa-Ramírez et al., 2023). High values of ΔSMO_2_AI and ΔSMO_2_AV show fast recovery and do not indicate any type of significant fatigue. Post-training SMO_2_ values are usually higher than pre-training SMO_2_ values, which suggests a possible value of RMT as an active recovery protocol.

### Other measured variables

TL produced by RMT was assessed in the context of overall TL. Despite noting no significant statistical differences between training methods, sex, and time we found important practical differences. As mentioned earlier, the main sRPE contributor during RMT is its duration. Therefore, after the introduction period, with increased session length, VIH tends to generate higher sRPE week scores and higher %TTL compared to IPTL. Whereas %TTL calculated for IPTL is in 1.69%–2.29% range for the whole studied period, %TTL calculated for VIH for fourth week is 3.52% and 5.14% for males and females respectively, and for sixth week is 3.50% and 6.61% for males and females respectively. In our opinion, the mentioned values are noticeable and must be taken into account during training programming to establish an adequate level of physiological work and limit the risk of overreaching or overtraining. Suggestions that RMT sessions develop IMF may be found in the literature (Briskey et al., 2020; Iqbal et al., 2023). However, both the implemented RMT protocols and the methods of IMF assessment were different in each study. The findings of Briskey et al. (2020) indicate that systemic oxidative stress, as indicated by increased plasma F2-isoprostanes, occurs only during strenuous inspiratory flow-resistive breathing. Interestingly, the stress response was not associated with a decrease in transdiaphragmatic twitch pressures (Briskey et al., 2020). Iqbal et al. (2023) investigated the response of biomarkers, including fast skeletal troponin I, slow skeletal troponin I, creatine kinase-MB, fatty acid binding protein 3, myosin light chain 3, and myoglobin, in order to assess the presence of respiratory muscle damage in response to RMT. Although the presence of muscle damage was noted, the study informed more about the application of biomarker assessment of RMT-related muscle damage and fatigue, rather than the extent of the fatigue for different methods and loads of RMT (Iqbal et al., 2023). Moreover, the participants’ characteristics were different, since in both studies they were not highly-trained athletes and all the participants were males. There is no consensus on the percentage drop in measured values defined as IMF. However many studies use a 10% or 15% threshold in function decline (Luo et al., 2001; Mador et al., 2002) or statistically significant mean fall from baseline (McConnell et al., 1997; Lomax and McConnell, 2003). Therefore, assessing changes between pre- and post-RMT S-Index Tests, we did not observe RMT-induced IMF during our investigation independently on method, sex, and time. However, in many cases, the inspiratory muscle strength was improved post-RMT. Whereas the aforementioned studies investigated IMF in healthy subjects, only our study investigated IMF in well-trained athletes. Knowing that characteristics of inspiratory muscle strength depend on training level (Klusiewicz et al., 2014), we speculate that also the magnitude of IMF following RMT sessions may depend on the training status, fitness level, and pulmonary function level. Therefore, the investigated RMT protocols may potentially serve as an efficient respiratory warm-up in well-trained triathletes. Our finding also provides scientific background to the recommendations of Shei et al. about moving beyond a “One-Size Fits All” approach to RMT (Shei et al., 2022). The introduction of individualized training protocols that match athletes’ level of performance and competition demands should be the next step in developing RMT. We speculate that after the initial adaptation period, well-trained athletes may require a larger RMT stimulus to provide desirable adaptation, compared to less athletic populations. However, with pulmonology patients (Gosselink et al., 2011) or athletes recovering from illnesses affecting cardiopulmonary performance (Śliż et al., 2022; 2023) the same mechanism indicates that smaller RMT dose may elicit desirable adaptations.

### Strengths and limitations

The study investigated a homogeneous group of well-trained athletes. Therefore, the findings should not be extrapolated to different populations, such as sedentary subjects or patients. Despite the required sample size, relatively low number of participants may be considered a study limitation. Therefore, a replication study is required for the confirmation of the findings. Moreover, lack of sham-control group and spirometry measurements may be considered as a study limitations. Measurement of IMF was based on maximal maneuvers. Although this approach may be found in the literature, it requires a high level of participants’ motivation and therefore is prone to associated limitations. We acknowledge that phrenic nerve stimulation to assess diaphragmatic fatigue should be a valuable contribution to the study design, as it minimizes the influence of central nervous system and participants’ motivation on the IMF assessment. Response to RMT may depend on swim training characteristics in terms of both performance and induced stress. We did not monitor the swim training load in study participants, which may be an important consideration for their responses to RMT, as respiratory muscle adaptation to swim training is dose-dependent (Lomax et al., 2019). Novelty, larger female participation, applying the wide range of measurement methods and blinding the data analysts and laboratory technicians may be considered strengths of the study.

### Recommendations for further research

TL induced by RMT may differ depending on the chosen training method and investigated populations. Further research with different RMT methods and activity profiles, physical fitness, age, race, sex, and health status of participants are required. Specifically, the performance-oriented studies on RMT application in swimmers indicate that swim training may already be a significant stimulus for developing endurance and strength of respiratory muscles (Shei, 2018). Therefore, even in well-trained athletes there may be differences in RMT-induced stress and TL between populations that swim and do not swim in training or between athletes with different swimming TL. Moreover, with the advancement of training individualization, coaches and practitioners can offer athletes personalized RMT related to their individual profiles and sport-specific performance determinants. Hence, research should be carried out to develop measurement protocols that can effectively assess the individual TL induced by RMT and its influence on athletes.

## Conclusion

RMT induced larger changes in blood gasometry and cardiac indices in females rather than in males. VIH induced additional training load in well-trained triathletes. Despite the traditional objective indices such as T, C, and bLa concentrations, changes in blood gasometry, HR and local SMO_2_ did not suggest that VIH is a significant source of load for well-trained athletes, %TTL based on subjective assessments suggested that VIH was a relevant component of the training program and substantially contributes to overall TL. On the other hand, IPTL was associated with a disbalance in blood gasometry variables, an increase in bLa, and reports of headaches and dizziness. Although, the subjective assessments suggested the relatively low perceived impact of IPTL on well-trained athletes. Both methods of RMT should be applied with consideration, especially in the context of demanding training programs and athletes already training close to their personal capabilities. In such scenarios, common in high-performance environments, a few percent increase in TL might lead to excessive levels of fatigue, unnecessary risk of injury, or overtraining.

## Data Availability

The raw data supporting the conclusions of this article will be made available by the authors, without undue reservation.
