# Supplementary material for: Respiratory muscle training induces additional stress and training load in well-trained triathletes—randomized controlled trial
Source: Front Physiol. 2023 Sep 28;14:1264265. doi: 10.3389/fphys.2023.1264265 (PMC10576561; doi:10.3389/fphys.2023.1264265)
Supplement: Supplementary file 1 [file DataSheet1.pdf]

Supplementary Material

Respiratory muscle training induces additional stress and training load in well-trained triathletes - randomized controlled trial

Kowalski Tomasz[1*], Kasiak Przemysław Seweryn[2*], Rebis Kinga[1], Klusiewicz Andrzej[3], Granda Dominika[4], Wiecha Szczepan[3]

[1]Department of Physiology, Institute of Sport - National Research Institute, Warsaw, Poland

[2]3rd Department of Internal Medicine and Cardiology, Medical University of Warsaw, Warsaw, Poland

[3]Department of Physical Education and Health in Biala Podlaska, Jozef Pilsudski University of Physical Education, Faculty in Biala Podlaska, Poland

[4]Department of Nutrition Physiology and Dietetics, Institute of Sport - National Research Institute, Warsaw, Poland

***Correspondence:**

Kowalki Tomasz

tomasz.kowalski@insp.pl

Przemysław Seweryn Kasiak
przemyslaw.kasiak@wum.edu.pl

Reporting checklist for randomised trial.

Based on the CONSORT guidelines.

Schulz KF, Altman DG, Moher D, for the CONSORT Group. CONSORT 2010 Statement: updated guidelines for reporting parallel group randomised trials

		Reporting Item	
**Title and Abstract**			
Title	#1a	Identification as a randomized trial in the title.	✓
Abstract	#1b	Structured summary of trial design, methods, results, and conclusions	✓
**Introduction**			
Background and objectives	#2a	Scientific background and explanation of rationale	✓
Background and objectives	#2b	Specific objectives or hypothesis	✓
**Methods**			
Trial design	#3a	Description of trial design (such as parallel, factorial) including allocation ratio.	✓
Trial design	#3b	Important changes to methods after trial commencement (such as eligibility criteria), with reasons	N/A
Participants	#4a	Eligibility criteria for participants	✓
Participants	#4b	Settings and locations where the data were collected	✓
Interventions	#5	The experimental and control interventions for each group with sufficient details to allow replication, including how and when they were actually administered	✓
Outcomes	#6a	Completely defined prespecified primary and secondary outcome measures, including how and when they were assessed	✓

Sample size	#7a	How sample size was determined.	✓
Sample size	#7b	When applicable, explanation of any interim analyses and stopping guidelines	N/A
Randomization - Sequence generation	#8a	Method used to generate the random allocation sequence.	
3			
Randomization - Sequence generation	#8b	Type of randomization; details of any restriction (such as blocking and block size)	
3			
Randomization - Allocation concealment mechanism	#9	Mechanism used to implement the random allocation sequence (such as sequentially numbered containers), describing any steps taken to conceal the sequence until interventions were assigned	✓
Randomization - Implementation	#10	Who generated the allocation sequence, who enrolled participants, and who assigned participants to interventions	✓
Blinding	#11a	If done, who was blinded after assignment to interventions (for example, participants, care providers, those assessing outcomes) and how.	✓
Blinding	#11b	If relevant, description of the similarity of interventions	N/A
Statistical methods	#12a	Statistical methods used to compare groups for primary and secondary outcomes	✓
Statistical methods	#12b	Methods for additional analyses, such as subgroup analyses and adjusted analyses	N/A
Outcomes	#6b	Any changes to trial outcomes after the trial commenced, with reasons	N/A
**Results**			
Participant flow diagram (strongly recommended)	#13a	For each group, the numbers of participants who were randomly assigned, received intended treatment, and were analysed for the primary outcome	✓

Participant flow	#13b	For each group, losses and exclusions after randomization, together with reason	N/A
Recruitment	#14a	Dates defining the periods of recruitment and follow-up	✓
Recruitment	#14b	Why the trial ended or was stopped	N/A
Baseline data	#15	A table showing baseline demographic and clinical characteristics for each group	✓
Numbers analysed	#16	For each group, number of participants (denominator) included in each analysis and whether the analysis was by original assigned groups	✓
Outcomes and estimation	#17a	For each primary and secondary outcome, results for each group, and the estimated effect size and its precision (such as 95% confidence interval)	✓
Outcomes and estimation	#17b	For binary outcomes, presentation of both absolute and relative effect sizes is recommended	N/A
Ancillary analyses	#18	Results of any other analyses performed, including subgroup analyses and adjusted analyses, distinguishing pre-specified from exploratory	N/A
Harms	#19	All important harms or unintended effects in each group (For specific guidance see CONSORT for harms)	N/A
**Discussion**			
Limitations	#20	Trial limitations, addressing sources of potential bias, imprecision, and, if relevant, multiplicity of analyses	✓
Interpretation	#22	Interpretation consistent with results, balancing benefits and harms, and considering other relevant evidence	✓
Registration	#23	Registration number and name of trial registry	✓
Generalisability	#21	Generalisability (external validity, applicability) of the trial findings	✓
**Other information**			
Interpretation	#22	Interpretation consistent with results, balancing benefits and	✓

		harms, and considering other relevant evidence	
Registration	#23	Registration number and name of trial registry	✓
Protocol	#24	Where the full trial protocol can be accessed, if available	N/A
Funding	#25	Sources of funding and other support (such as supply of drugs), role of funders	✓

The CONSORT checklist is distributed under the terms of the Creative Commons Attribution License CC-BY.
